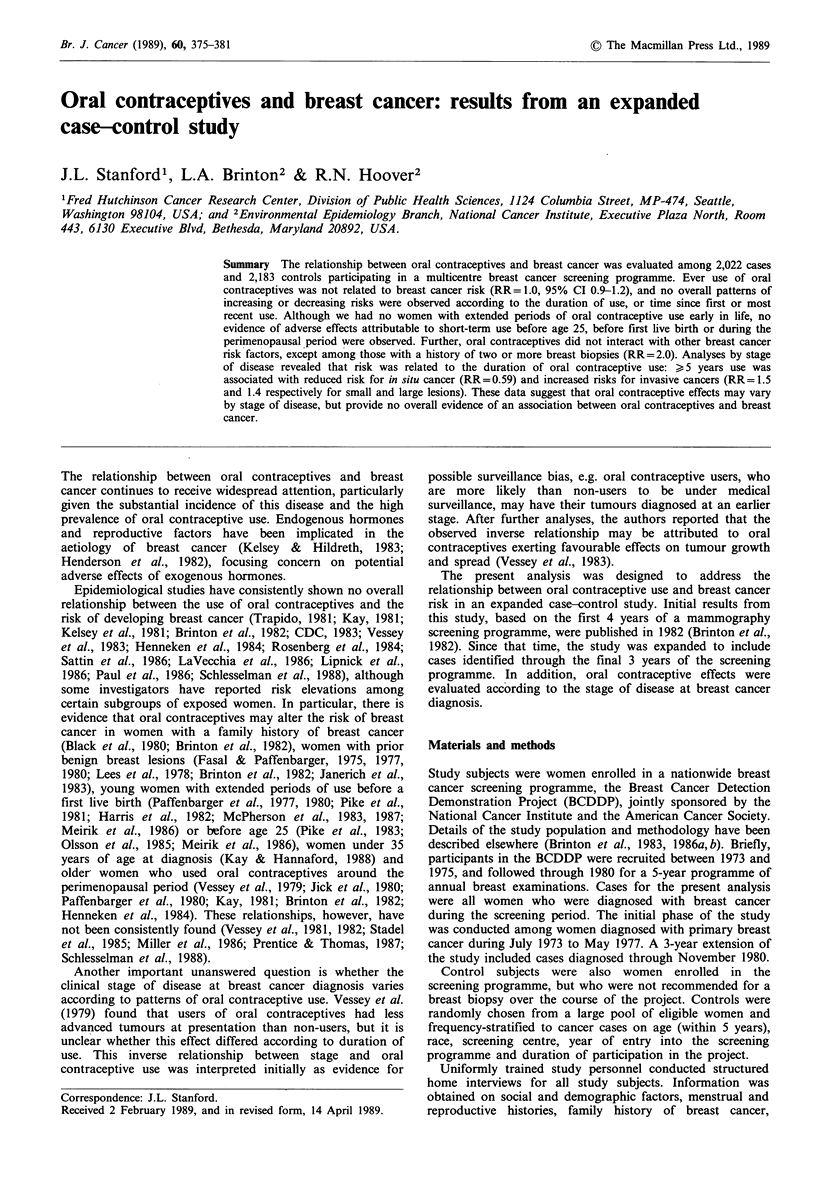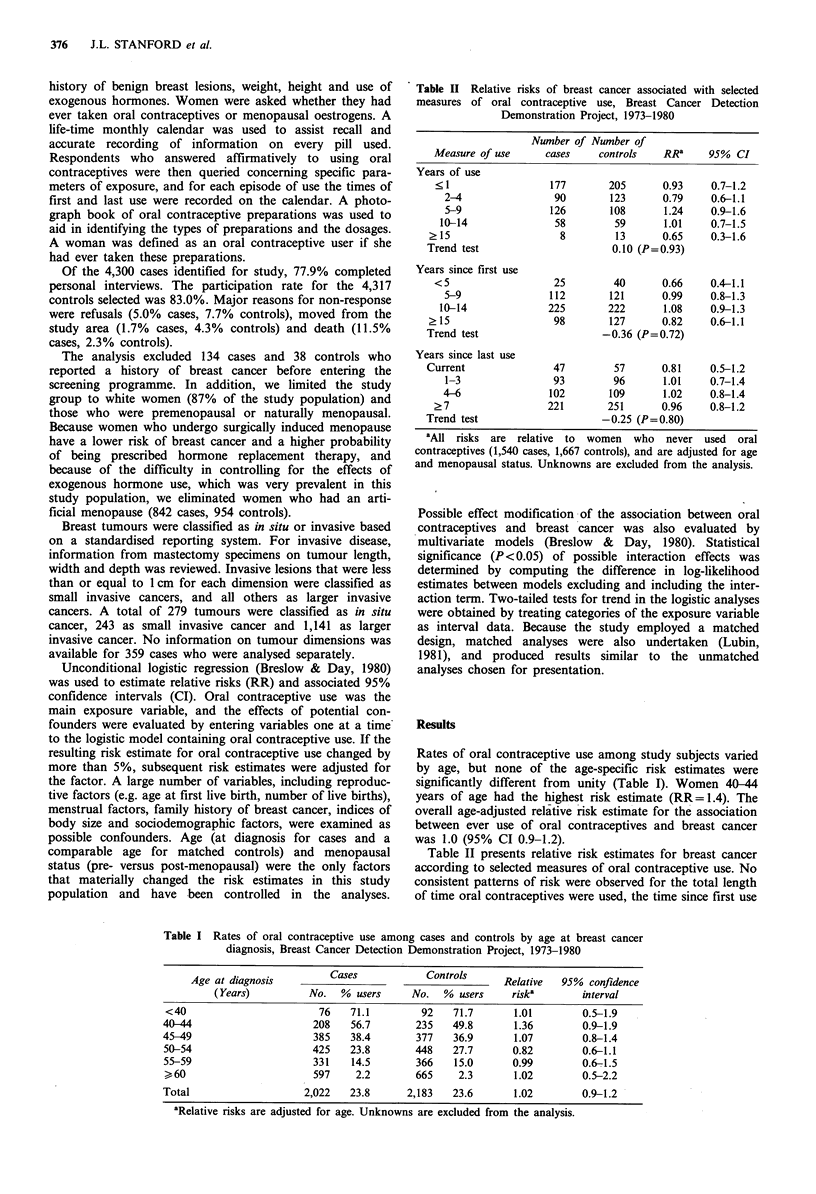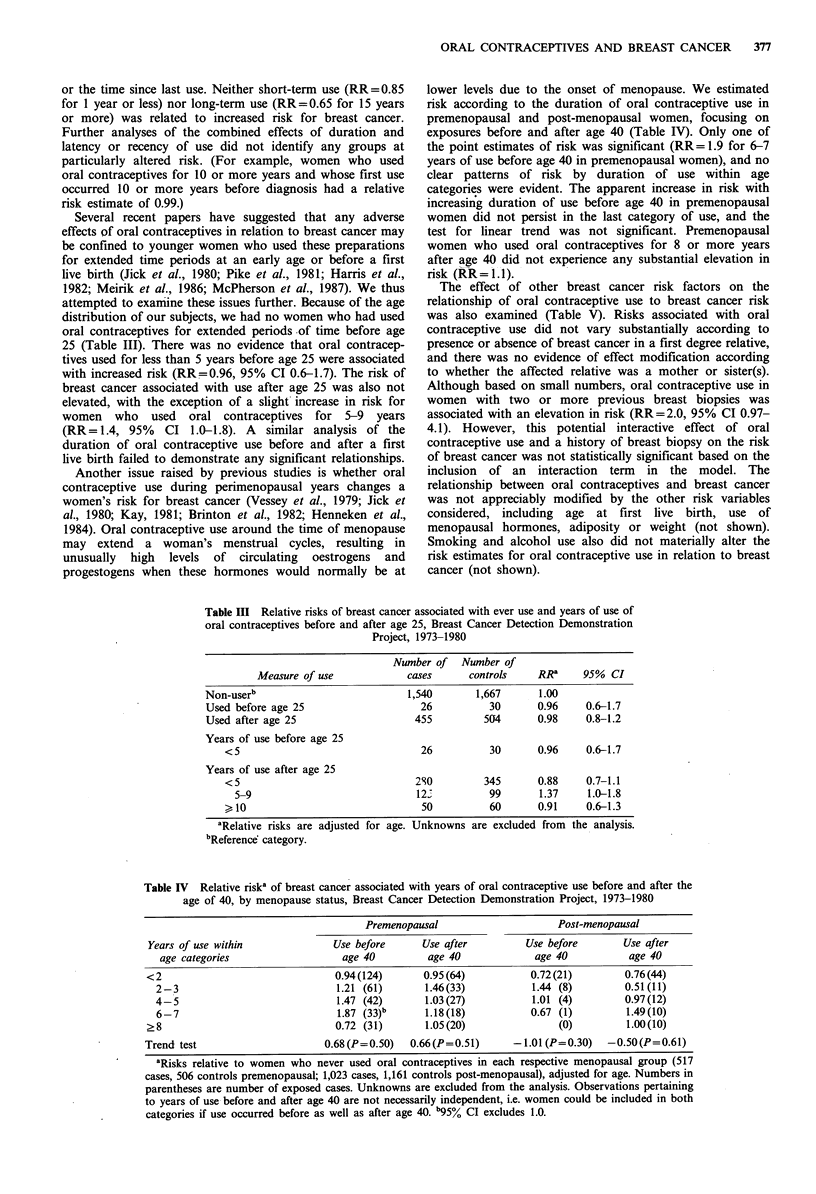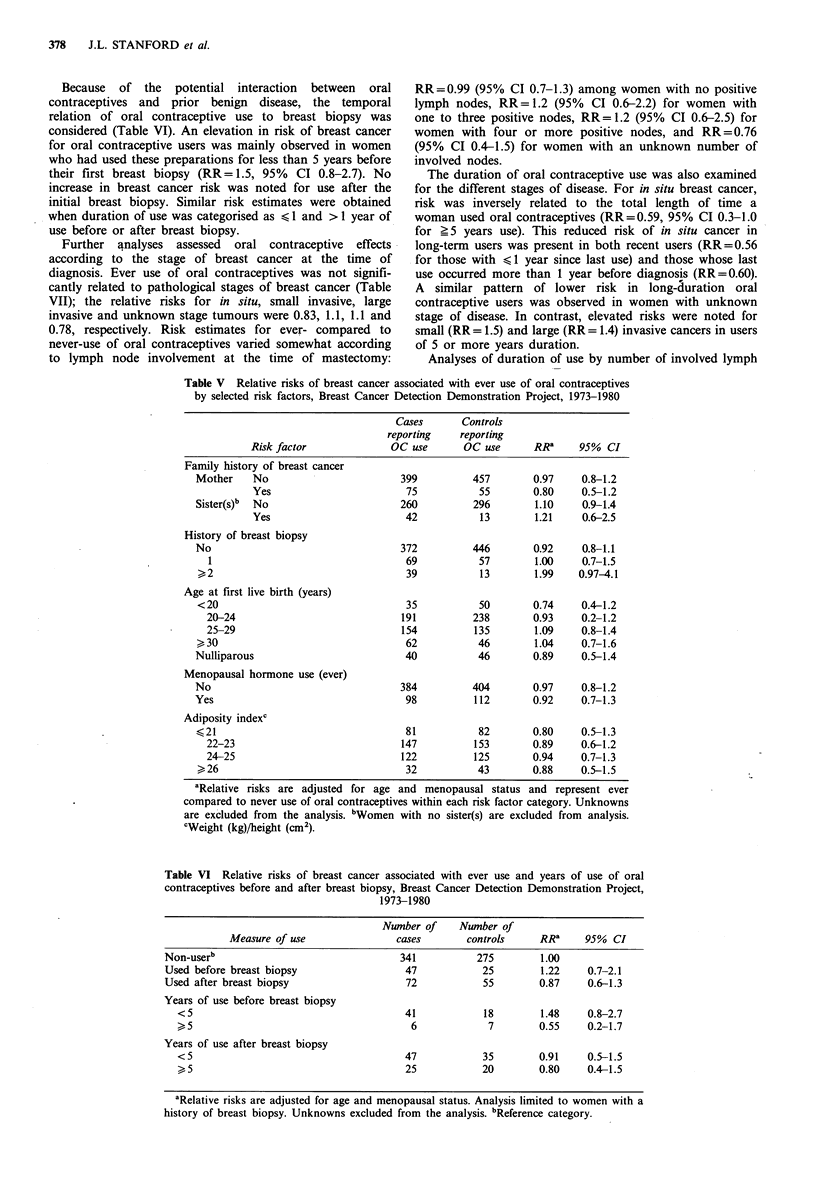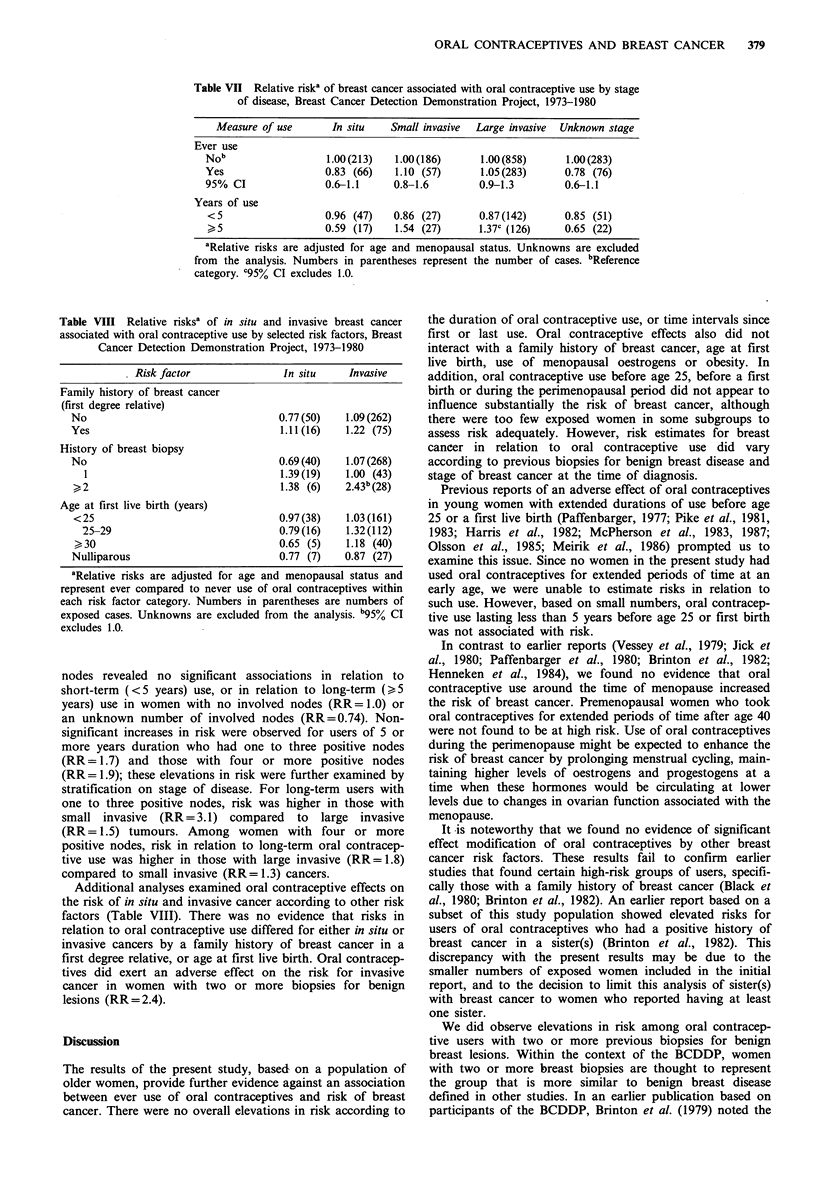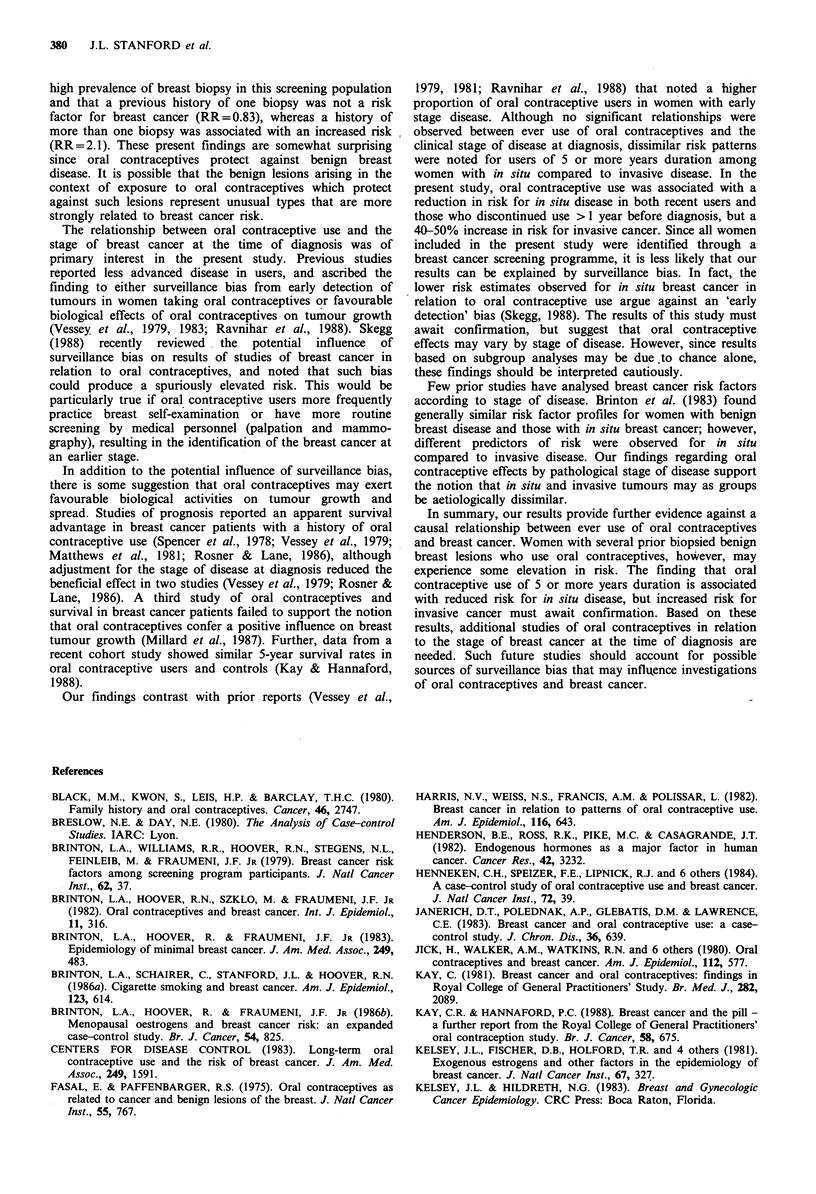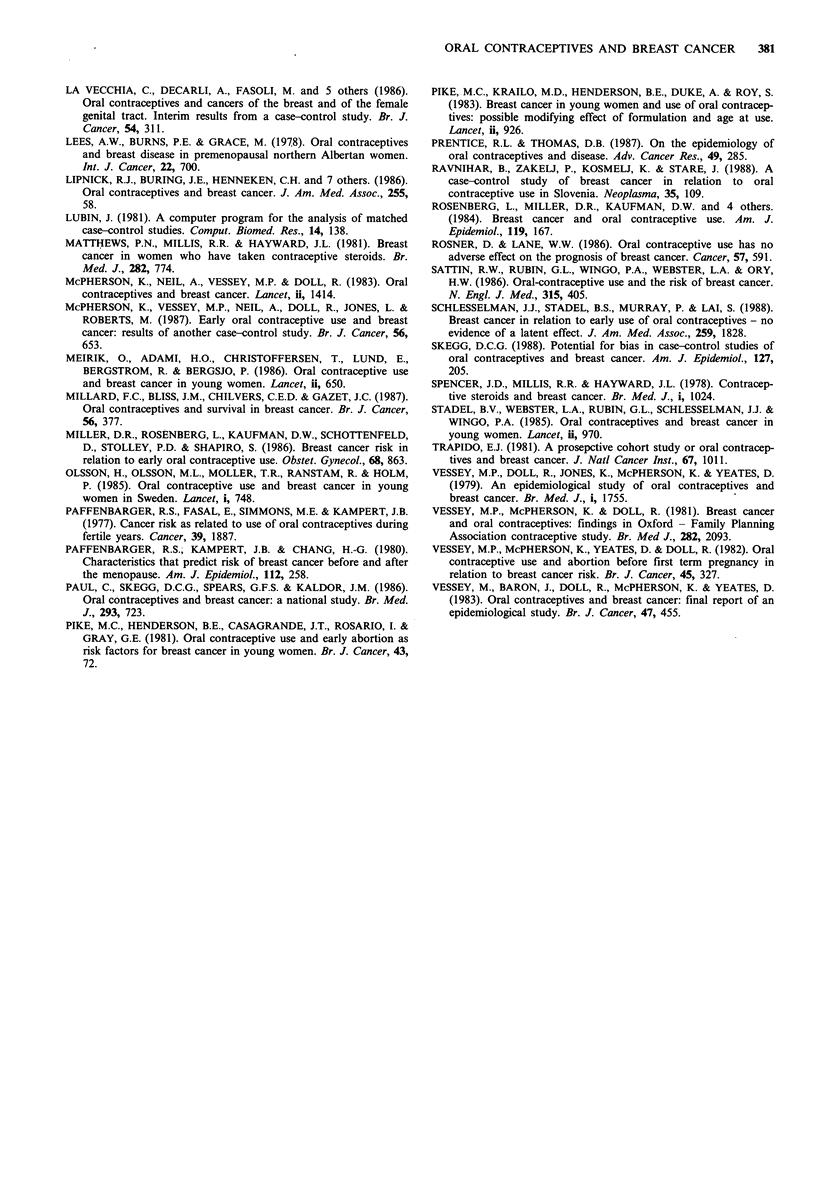# Oral contraceptives and breast cancer: results from an expanded case-control study.

**DOI:** 10.1038/bjc.1989.288

**Published:** 1989-09

**Authors:** J. L. Stanford, L. A. Brinton, R. N. Hoover

**Affiliations:** Fred Hutchinson Cancer Research Center, Division of Public Health Sciences, Seattle, Washington 98104.

## Abstract

The relationship between oral contraceptives and breast cancer was evaluated among 2,022 cases and 2,183 controls participating in a multicentre breast cancer screening programme. Ever use of oral contraceptives was not related to breast cancer risk (RR = 1.0, 95% CI 0.9-1.2), and no overall patterns of increasing or decreasing risks were observed according to the duration of use, or time since first or most recent use. Although we had no women with extended periods of oral contraceptive use early in life, no evidence of adverse effects attributable to short-term use before age 25, before first live birth or during the perimenopausal period were observed. Further, oral contraceptives did not interact with other breast cancer risk factors, except among those with a history of two or more breast biopsies (RR = 2.0). Analyses by stage of disease revealed that risk was related to the duration of oral contraceptive use: greater than or equal to 5 years use was associated with reduced risk for in situ cancer (RR = 0.59) and increased risks for invasive cancers (RR = 1.5 and 1.4 respectively for small and large lesions). These data suggest that oral contraceptive effects may vary by stage of disease, but provide no overall evidence of an association between oral contraceptives and breast cancer.


*Br. J. Cancer* (1989), **60**, 375–381 © The Macmillan Press Ltd., 1989

### Oral contraceptives and breast cancer: results from an expanded case–control study

J.L. Stanford[1], L.A. Brinton[2] & R.N. Hoover[2]

[1]*Fred Hutchinson Cancer Research Center, Division of Public Health Sciences, 1124 Columbia Street, MP-474, Seattle, Washington 98104, USA; and* [2]*Environmental Epidemiology Branch, National Cancer Institute, Executive Plaza North, Room 443, 6130 Executive Blvd, Bethesda, Maryland 20892, USA.*

**Summary** The relationship between oral contraceptives and breast cancer was evaluated among 2,022 cases and 2,183 controls participating in a multicentre breast cancer screening programme. Ever use of oral contraceptives was not related to breast cancer risk (RR = 1.0, 95% CI 0.9–1.2), and no overall patterns of increasing or decreasing risks were observed according to the duration of use, or time since first or most recent use. Although we had no women with extended periods of oral contraceptive use early in life, no evidence of adverse effects attributable to short-term use before age 25, before first live birth or during the perimenopausal period were observed. Further, oral contraceptives did not interact with other breast cancer risk factors, except among those with a history of two or more breast biopsies (RR = 2.0). Analyses by stage of disease revealed that risk was related to the duration of oral contraceptive use: ⩾5 years use was associated with reduced risk for *in situ* cancer (RR = 0.59) and increased risks for invasive cancers (RR = 1.5 and 1.4 respectively for small and large lesions). These data suggest that oral contraceptive effects may vary by stage of disease, but provide no overall evidence of an association between oral contraceptives and breast cancer.

The relationship between oral contraceptives and breast cancer continues to receive widespread attention, particularly given the substantial incidence of this disease and the high prevalence of oral contraceptive use. Endogenous hormones and reproductive factors have been implicated in the aetiology of breast cancer (Kelsey & Hildreth, 1983; Henderson *et al.*, 1982), focusing concern on potential adverse effects of exogenous hormones.

Epidemiological studies have consistently shown no overall relationship between the use of oral contraceptives and the risk of developing breast cancer (Trapido, 1981; Kay, 1981; Kelsey *et al.*, 1981; Brinton *et al.*, 1982; CDC, 1983; Vessey *et al.*, 1983; Henneken *et al.*, 1984; Rosenberg *et al.*, 1984; Sattin *et al.*, 1986; LaVecchia *et al.*, 1986; Lipnick *et al.*, 1986; Paul *et al.*, 1986; Schlesselman *et al.*, 1988), although some investigators have reported risk elevations among certain subgroups of exposed women. In particular, there is evidence that oral contraceptives may alter the risk of breast cancer in women with a family history of breast cancer (Black *et al.*, 1980; Brinton *et al.*, 1982), women with prior benign breast lesions (Fasal & Paffenbarger, 1975, 1977, 1980; Lees *et al.*, 1978; Brinton *et al.*, 1982; Janerich *et al.*, 1983), young women with extended periods of use before a first live birth (Paffenbarger *et al.*, 1977, 1980; Pike *et al.*, 1981; Harris *et al.*, 1982; McPherson *et al.*, 1983, 1987; Meirik *et al.*, 1986) or before age 25 (Pike *et al.*, 1983; Olsson *et al.*, 1985; Meirik *et al.*, 1986), women under 35 years of age at diagnosis (Kay & Hannaford, 1988) and older women who used oral contraceptives around the perimenopausal period (Vessey *et al.*, 1979; Jick *et al.*, 1980; Paffenbarger *et al.*, 1980; Kay, 1981; Brinton *et al.*, 1982; Henneken *et al.*, 1984). These relationships, however, have not been consistently found (Vessey *et al.*, 1981, 1982; Stadel *et al.*, 1985; Miller *et al.*, 1986; Prentice & Thomas, 1987; Schlesselman *et al.*, 1988).

Another important unanswered question is whether the clinical stage of disease at breast cancer diagnosis varies according to patterns of oral contraceptive use. Vessey *et al.* (1979) found that users of oral contraceptives had less advanced tumours at presentation than non-users, but it is unclear whether this effect differed according to duration of use. This inverse relationship between stage and oral contraceptive use was interpreted initially as evidence for possible surveillance bias, e.g. oral contraceptive users, who are more likely than non-users to be under medical surveillance, may have their tumours diagnosed at an earlier stage. After further analyses, the authors reported that the observed inverse relationship may be attributed to oral contraceptives exerting favourable effects on tumour growth and spread (Vessey *et al.*, 1983).

The present analysis was designed to address the relationship between oral contraceptive use and breast cancer risk in an expanded case–control study. Initial results from this study, based on the first 4 years of a mammography screening programme, were published in 1982 (Brinton *et al.*, 1982). Since that time, the study was expanded to include cases identified through the final 3 years of the screening programme. In addition, oral contraceptive effects were evaluated according to the stage of disease at breast cancer diagnosis.

### Materials and methods

Study subjects were women enrolled in a nationwide breast cancer screening programme, the Breast Cancer Detection Demonstration Project (BCDDP), jointly sponsored by the National Cancer Institute and the American Cancer Society. Details of the study population and methodology have been described elsewhere (Brinton *et al.*, 1983, 1986*a*, *b*). Briefly, participants in the BCDDP were recruited between 1973 and 1975, and followed through 1980 for a 5-year programme of annual breast examinations. Cases for the present analysis were all women who were diagnosed with breast cancer during the screening period. The initial phase of the study was conducted among women diagnosed with primary breast cancer during July 1973 to May 1977. A 3-year extension of the study included cases diagnosed through November 1980.

Control subjects were also women enrolled in the screening programme, but who were not recommended for a breast biopsy over the course of the project. Controls were randomly chosen from a large pool of eligible women and frequency-stratified to cancer cases on age (within 5 years), race, screening centre, year of entry into the screening programme and duration of participation in the project.

Uniformly trained study personnel conducted structured home interviews for all study subjects. Information was obtained on social and demographic factors, menstrual and reproductive histories, family history of breast cancer,

Correspondence: J.L. Stanford.
Received 2 February 1989, and in revised form, 14 April 1989.

history of benign breast lesions, weight, height and use of exogenous hormones. Women were asked whether they had ever taken oral contraceptives or menopausal oestrogens. A life-time monthly calendar was used to assist recall and accurate recording of information on every pill used. Respondents who answered affirmatively to using oral contraceptives were then queried concerning specific parameters of exposure, and for each episode of use the times of first and last use were recorded on the calendar. A photograph book of oral contraceptive preparations was used to aid in identifying the types of preparations and the dosages. A woman was defined as an oral contraceptive user if she had ever taken these preparations.

Of the 4,300 cases identified for study, 77.9% completed personal interviews. The participation rate for the 4,317 controls selected was 83.0%. Major reasons for non-response were refusals (5.0% cases, 7.7% controls), moved from the study area (1.7% cases, 4.3% controls) and death (11.5% cases, 2.3% controls).

The analysis excluded 134 cases and 38 controls who reported a history of breast cancer before entering the screening programme. In addition, we limited the study group to white women (87% of the study population) and those who were premenopausal or naturally menopausal. Because women who undergo surgically induced menopause have a lower risk of breast cancer and a higher probability of being prescribed hormone replacement therapy, and because of the difficulty in controlling for the effects of exogenous hormone use, which was very prevalent in this study population, we eliminated women who had an artificial menopause (842 cases, 954 controls).

Breast tumours were classified as *in situ* or invasive based on a standardised reporting system. For invasive disease, information from mastectomy specimens on tumour length, width and depth was reviewed. Invasive lesions that were less than or equal to 1 cm for each dimension were classified as small invasive cancers, and all others as larger invasive cancers. A total of 279 tumours were classified as *in situ* cancer, 243 as small invasive cancer and 1,141 as larger invasive cancer. No information on tumour dimensions was available for 359 cases who were analysed separately.

Unconditional logistic regression (Breslow & Day, 1980) was used to estimate relative risks (RR) and associated 95% confidence intervals (CI). Oral contraceptive use was the main exposure variable, and the effects of potential confounders were evaluated by entering variables one at a time to the logistic model containing oral contraceptive use. If the resulting risk estimate for oral contraceptive use changed by more than 5%, subsequent risk estimates were adjusted for the factor. A large number of variables, including reproductive factors (e.g. age at first live birth, number of live births), menstrual factors, family history of breast cancer, indices of body size and sociodemographic factors, were examined as possible confounders. Age (at diagnosis for cases and a comparable age for matched controls) and menopausal status (pre- versus post-menopausal) were the only factors that materially changed the risk estimates in this study population and have been controlled in the analyses.

**Table II** Relative risks of breast cancer associated with selected measures of oral contraceptive use, Breast Cancer Detection Demonstration Project, 1973–1980

| *Measure of use* | *Number of cases* | *Number of controls* | *RR*[a] | *95% CI* |
|---|---|---|---|---|
| Years of use | | | | |
| ≤1 | 177 | 205 | 0.93 | 0.7–1.2 |
| 2–4 | 90 | 123 | 0.79 | 0.6–1.1 |
| 5–9 | 126 | 108 | 1.24 | 0.9–1.6 |
| 10–14 | 58 | 59 | 1.01 | 0.7–1.5 |
| ≥15 | 8 | 13 | 0.65 | 0.3–1.6 |
| Trend test | | 0.10 ($P$=0.93) | | |
| Years since first use | | | | |
| <5 | 25 | 40 | 0.66 | 0.4–1.1 |
| 5–9 | 112 | 121 | 0.99 | 0.8–1.3 |
| 10–14 | 225 | 222 | 1.08 | 0.9–1.3 |
| ≥15 | 98 | 127 | 0.82 | 0.6–1.1 |
| Trend test | | −0.36 ($P$=0.72) | | |
| Years since last use | | | | |
| Current | 47 | 57 | 0.81 | 0.5–1.2 |
| 1–3 | 93 | 96 | 1.01 | 0.7–1.4 |
| 4–6 | 102 | 109 | 1.02 | 0.8–1.4 |
| ≥7 | 221 | 251 | 0.96 | 0.8–1.2 |
| Trend test | | −0.25 ($P$=0.80) | | |

[a]All risks are relative to women who never used oral contraceptives (1,540 cases, 1,667 controls), and are adjusted for age and menopausal status. Unknowns are excluded from the analysis.

Possible effect modification of the association between oral contraceptives and breast cancer was also evaluated by multivariate models (Breslow & Day, 1980). Statistical significance ($P<0.05$) of possible interaction effects was determined by computing the difference in log-likelihood estimates between models excluding and including the interaction term. Two-tailed tests for trend in the logistic analyses were obtained by treating categories of the exposure variable as interval data. Because the study employed a matched design, matched analyses were also undertaken (Lubin, 1981), and produced results similar to the unmatched analyses chosen for presentation.

### Results

Rates of oral contraceptive use among study subjects varied by age, but none of the age-specific risk estimates were significantly different from unity (Table I). Women 40–44 years of age had the highest risk estimate (RR = 1.4). The overall age-adjusted relative risk estimate for the association between ever use of oral contraceptives and breast cancer was 1.0 (95% CI 0.9–1.2).

Table II presents relative risk estimates for breast cancer according to selected measures of oral contraceptive use. No consistent patterns of risk were observed for the total length of time oral contraceptives were used, the time since first use

**Table I** Rates of oral contraceptive use among cases and controls by age at breast cancer diagnosis, Breast Cancer Detection Demonstration Project, 1973–1980

| *Age at diagnosis (Years)* | *Cases* | | *Controls* | | *Relative risk*[a] | *95% confidence interval* |
|---|---|---|---|---|---|---|
| | *No.* | *% users* | *No.* | *% users* | | |
| <40 | 76 | 71.1 | 92 | 71.7 | 1.01 | 0.5–1.9 |
| 40–44 | 208 | 56.7 | 235 | 49.8 | 1.36 | 0.9–1.9 |
| 45–49 | 385 | 38.4 | 377 | 36.9 | 1.07 | 0.8–1.4 |
| 50–54 | 425 | 23.8 | 448 | 27.7 | 0.82 | 0.6–1.1 |
| 55–59 | 331 | 14.5 | 366 | 15.0 | 0.99 | 0.6–1.5 |
| ⩾60 | 597 | 2.2 | 665 | 2.3 | 1.02 | 0.5–2.2 |
| Total | 2,022 | 23.8 | 2,183 | 23.6 | 1.02 | 0.9–1.2 |

[a]Relative risks are adjusted for age. Unknowns are excluded from the analysis.

or the time since last use. Neither short-term use (RR = 0.85 for 1 year or less) nor long-term use (RR = 0.65 for 15 years or more) was related to increased risk for breast cancer. Further analyses of the combined effects of duration and latency or recency of use did not identify any groups at particularly altered risk. (For example, women who used oral contraceptives for 10 or more years and whose first use occurred 10 or more years before diagnosis had a relative risk estimate of 0.99.)

Several recent papers have suggested that any adverse effects of oral contraceptives in relation to breast cancer may be confined to younger women who used these preparations for extended time periods at an early age or before a first live birth (Jick *et al.*, 1980; Pike *et al.*, 1981; Harris *et al.*, 1982; Meirik *et al.*, 1986; McPherson *et al.*, 1987). We thus attempted to examine these issues further. Because of the age distribution of our subjects, we had no women who had used oral contraceptives for extended periods of time before age 25 (Table III). There was no evidence that oral contraceptives used for less than 5 years before age 25 were associated with increased risk (RR = 0.96, 95% CI 0.6–1.7). The risk of breast cancer associated with use after age 25 was also not elevated, with the exception of a slight increase in risk for women who used oral contraceptives for 5–9 years (RR = 1.4, 95% CI 1.0–1.8). A similar analysis of the duration of oral contraceptive use before and after a first live birth failed to demonstrate any significant relationships.

Another issue raised by previous studies is whether oral contraceptive use during perimenopausal years changes a women's risk for breast cancer (Vessey *et al.*, 1979; Jick *et al.*, 1980; Kay, 1981; Brinton *et al.*, 1982; Henneken *et al.*, 1984). Oral contraceptive use around the time of menopause may extend a woman's menstrual cycles, resulting in unusually high levels of circulating oestrogens and progestogens when these hormones would normally be at lower levels due to the onset of menopause. We estimated risk according to the duration of oral contraceptive use in premenopausal and post-menopausal women, focusing on exposures before and after age 40 (Table IV). Only one of the point estimates of risk was significant (RR = 1.9 for 6–7 years of use before age 40 in premenopausal women), and no clear patterns of risk by duration of use within age categories were evident. The apparent increase in risk with increasing duration of use before age 40 in premenopausal women did not persist in the last category of use, and the test for linear trend was not significant. Premenopausal women who used oral contraceptives for 8 or more years after age 40 did not experience any substantial elevation in risk (RR = 1.1).

The effect of other breast cancer risk factors on the relationship of oral contraceptive use to breast cancer risk was also examined (Table V). Risks associated with oral contraceptive use did not vary substantially according to presence or absence of breast cancer in a first degree relative, and there was no evidence of effect modification according to whether the affected relative was a mother or sister(s). Although based on small numbers, oral contraceptive use in women with two or more previous breast biopsies was associated with an elevation in risk (RR = 2.0, 95% CI 0.97–4.1). However, this potential interactive effect of oral contraceptive use and a history of breast biopsy on the risk of breast cancer was not statistically significant based on the inclusion of an interaction term in the model. The relationship between oral contraceptives and breast cancer was not appreciably modified by the other risk variables considered, including age at first live birth, use of menopausal hormones, adiposity or weight (not shown). Smoking and alcohol use also did not materially alter the risk estimates for oral contraceptive use in relation to breast cancer (not shown).

**Table III** Relative risks of breast cancer associated with ever use and years of use of oral contraceptives before and after age 25, Breast Cancer Detection Demonstration Project, 1973–1980

| *Measure of use* | *Number of cases* | *Number of controls* | *RR*[a] | *95% CI* |
|---|---|---|---|---|
| Non-user[b] | 1,540 | 1,667 | 1.00 | |
| Used before age 25 | 26 | 30 | 0.96 | 0.6–1.7 |
| Used after age 25 | 455 | 504 | 0.98 | 0.8–1.2 |
| Years of use before age 25 | | | | |
| <5 | 26 | 30 | 0.96 | 0.6–1.7 |
| Years of use after age 25 | | | | |
| <5 | 280 | 345 | 0.88 | 0.7–1.1 |
| 5–9 | 125 | 99 | 1.37 | 1.0–1.8 |
| ⩾10 | 50 | 60 | 0.91 | 0.6–1.3 |

[a]Relative risks are adjusted for age. Unknowns are excluded from the analysis. [b]Reference category.

**Table IV** Relative risk[a] of breast cancer associated with years of oral contraceptive use before and after the age of 40, by menopause status, Breast Cancer Detection Demonstration Project, 1973–1980

| | *Premenopausal* | | *Post-menopausal* | |
|---|---|---|---|---|
| *Years of use within age categories* | *Use before age 40* | *Use after age 40* | *Use before age 40* | *Use after age 40* |
| <2 | 0.94 (124) | 0.95 (64) | 0.72 (21) | 0.76 (44) |
| 2–3 | 1.21 (61) | 1.46 (33) | 1.44 (8) | 0.51 (11) |
| 4–5 | 1.47 (42) | 1.03 (27) | 1.01 (4) | 0.97 (12) |
| 6–7 | 1.87 (33)[b] | 1.18 (18) | 0.67 (1) | 1.49 (10) |
| ≥8 | 0.72 (31) | 1.05 (20) | (0) | 1.00 (10) |
| Trend test | 0.68 ($P$=0.50) | 0.66 ($P$=0.51) | −1.01 ($P$=0.30) | −0.50 ($P$=0.61) |

[a]Risks relative to women who never used oral contraceptives in each respective menopausal group (517 cases, 506 controls premenopausal; 1,023 cases, 1,161 controls post-menopausal), adjusted for age. Numbers in parentheses are number of exposed cases. Unknowns are excluded from the analysis. Observations pertaining to years of use before and after age 40 are not necessarily independent, i.e. women could be included in both categories if use occurred before as well as after age 40. [b]95% CI excludes 1.0.

Because of the potential interaction between oral contraceptives and prior benign disease, the temporal relation of oral contraceptive use to breast biopsy was considered (Table VI). An elevation in risk of breast cancer for oral contraceptive users was mainly observed in women who had used these preparations for less than 5 years before their first breast biopsy (RR = 1.5, 95% CI 0.8–2.7). No increase in breast cancer risk was noted for use after the initial breast biopsy. Similar risk estimates were obtained when duration of use was categorised as ⩽1 and >1 year of use before or after breast biopsy.

Further analyses assessed oral contraceptive effects according to the stage of breast cancer at the time of diagnosis. Ever use of oral contraceptives was not significantly related to pathological stages of breast cancer (Table VII); the relative risks for *in situ*, small invasive, large invasive and unknown stage tumours were 0.83, 1.1, 1.1 and 0.78, respectively. Risk estimates for ever- compared to never-use of oral contraceptives varied somewhat according to lymph node involvement at the time of mastectomy: RR = 0.99 (95% CI 0.7–1.3) among women with no positive lymph nodes, RR = 1.2 (95% CI 0.6–2.2) for women with one to three positive nodes, RR = 1.2 (95% CI 0.6–2.5) for women with four or more positive nodes, and RR = 0.76 (95% CI 0.4–1.5) for women with an unknown number of involved nodes.

The duration of oral contraceptive use was also examined for the different stages of disease. For *in situ* breast cancer, risk was inversely related to the total length of time a woman used oral contraceptives (RR = 0.59, 95% CI 0.3–1.0 for ≧5 years use). This reduced risk of *in situ* cancer in long-term users was present in both recent users (RR = 0.56 for those with ⩽1 year since last use) and those whose last use occurred more than 1 year before diagnosis (RR = 0.60). A similar pattern of lower risk in long-duration oral contraceptive users was observed in women with unknown stage of disease. In contrast, elevated risks were noted for small (RR = 1.5) and large (RR = 1.4) invasive cancers in users of 5 or more years duration.

Analyses of duration of use by number of involved lymph

**Table V** Relative risks of breast cancer associated with ever use of oral contraceptives by selected risk factors, Breast Cancer Detection Demonstration Project, 1973–1980

| *Risk factor* | *Cases reporting OC use* | *Controls reporting OC use* | *RR*[a] | *95% CI* |
|---|---|---|---|---|
| Family history of breast cancer | | | | |
| Mother No | 399 | 457 | 0.97 | 0.8–1.2 |
| Yes | 75 | 55 | 0.80 | 0.5–1.2 |
| Sister(s)[b] No | 260 | 296 | 1.10 | 0.9–1.4 |
| Yes | 42 | 13 | 1.21 | 0.6–2.5 |
| History of breast biopsy | | | | |
| No | 372 | 446 | 0.92 | 0.8–1.1 |
| 1 | 69 | 57 | 1.00 | 0.7–1.5 |
| ⩾2 | 39 | 13 | 1.99 | 0.97–4.1 |
| Age at first live birth (years) | | | | |
| <20 | 35 | 50 | 0.74 | 0.4–1.2 |
| 20–24 | 191 | 238 | 0.93 | 0.2–1.2 |
| 25–29 | 154 | 135 | 1.09 | 0.8–1.4 |
| ⩾30 | 62 | 46 | 1.04 | 0.7–1.6 |
| Nulliparous | 40 | 46 | 0.89 | 0.5–1.4 |
| Menopausal hormone use (ever) | | | | |
| No | 384 | 404 | 0.97 | 0.8–1.2 |
| Yes | 98 | 112 | 0.92 | 0.7–1.3 |
| Adiposity index[c] | | | | |
| ⩽21 | 81 | 82 | 0.80 | 0.5–1.3 |
| 22–23 | 147 | 153 | 0.89 | 0.6–1.2 |
| 24–25 | 122 | 125 | 0.94 | 0.7–1.3 |
| ⩾26 | 32 | 43 | 0.88 | 0.5–1.5 |

[a]Relative risks are adjusted for age and menopausal status and represent ever compared to never use of oral contraceptives within each risk factor category. Unknowns are excluded from the analysis. [b]Women with no sister(s) are excluded from analysis. [c]Weight (kg)/height ($cm^2$).

**Table VI** Relative risks of breast cancer associated with ever use and years of use of oral contraceptives before and after breast biopsy, Breast Cancer Detection Demonstration Project, 1973–1980

| *Measure of use* | *Number of cases* | *Number of controls* | *RR*[a] | *95% CI* |
|---|---|---|---|---|
| Non-user[b] | 341 | 275 | 1.00 | |
| Used before breast biopsy | 47 | 25 | 1.22 | 0.7–2.1 |
| Used after breast biopsy | 72 | 55 | 0.87 | 0.6–1.3 |
| Years of use before breast biopsy | | | | |
| <5 | 41 | 18 | 1.48 | 0.8–2.7 |
| ⩾5 | 6 | 7 | 0.55 | 0.2–1.7 |
| Years of use after breast biopsy | | | | |
| <5 | 47 | 35 | 0.91 | 0.5–1.5 |
| ⩾5 | 25 | 20 | 0.80 | 0.4–1.5 |

[a]Relative risks are adjusted for age and menopausal status. Analysis limited to women with a history of breast biopsy. Unknowns excluded from the analysis. [b]Reference category.

**Table VII** Relative risk[a] of breast cancer associated with oral contraceptive use by stage of disease, Breast Cancer Detection Demonstration Project, 1973–1980

| *Measure of use* | *In situ* | *Small invasive* | *Large invasive* | *Unknown stage* |
|---|---|---|---|---|
| Ever use | | | | |
| No[b] | 1.00 (213) | 1.00 (186) | 1.00 (858) | 1.00 (283) |
| Yes | 0.83 (66) | 1.10 (57) | 1.05 (283) | 0.78 (76) |
| 95% CI | 0.6–1.1 | 0.8–1.6 | 0.9–1.3 | 0.6–1.1 |
| Years of use | | | | |
| <5 | 0.96 (47) | 0.86 (27) | 0.87 (142) | 0.85 (51) |
| ⩾5 | 0.59 (17) | 1.54 (27) | 1.37[c] (126) | 0.65 (22) |

[a]Relative risks are adjusted for age and menopausal status. Unknowns are excluded from the analysis. Numbers in parentheses represent the number of cases. [b]Reference category. [c]95% CI excludes 1.0.

**Table VIII** Relative risks[a] of *in situ* and invasive breast cancer associated with oral contraceptive use by selected risk factors, Breast Cancer Detection Demonstration Project, 1973–1980

| *Risk factor* | *In situ* | *Invasive* |
|---|---|---|
| Family history of breast cancer (first degree relative) | | |
| No | 0.77 (50) | 1.09 (262) |
| Yes | 1.11 (16) | 1.22 (75) |
| History of breast biopsy | | |
| No | 0.69 (40) | 1.07 (268) |
| 1 | 1.39 (19) | 1.00 (43) |
| ⩾2 | 1.38 (6) | 2.43[b] (28) |
| Age at first live birth (years) | | |
| <25 | 0.97 (38) | 1.03 (161) |
| 25–29 | 0.79 (16) | 1.32 (112) |
| ⩾30 | 0.65 (5) | 1.18 (40) |
| Nulliparous | 0.77 (7) | 0.87 (27) |

[a]Relative risks are adjusted for age and menopausal status and represent ever compared to never use of oral contraceptives within each risk factor category. Numbers in parentheses are numbers of exposed cases. Unknowns are excluded from the analysis. [b]95% CI excludes 1.0.

nodes revealed no significant associations in relation to short-term (<5 years) use, or in relation to long-term (⩾5 years) use in women with no involved nodes (RR = 1.0) or an unknown number of involved nodes (RR = 0.74). Non-significant increases in risk were observed for users of 5 or more years duration who had one to three positive nodes (RR = 1.7) and those with four or more positive nodes (RR = 1.9); these elevations in risk were further examined by stratification on stage of disease. For long-term users with one to three positive nodes, risk was higher in those with small invasive (RR = 3.1) compared to large invasive (RR = 1.5) tumours. Among women with four or more positive nodes, risk in relation to long-term oral contraceptive use was higher in those with large invasive (RR = 1.8) compared to small invasive (RR = 1.3) cancers.

Additional analyses examined oral contraceptive effects on the risk of *in situ* and invasive cancer according to other risk factors (Table VIII). There was no evidence that risks in relation to oral contraceptive use differed for either *in situ* or invasive cancers by a family history of breast cancer in a first degree relative, or age at first live birth. Oral contraceptives did exert an adverse effect on the risk for invasive cancer in women with two or more biopsies for benign lesions (RR = 2.4).

### Discussion

The results of the present study, based on a population of older women, provide further evidence against an association between ever use of oral contraceptives and risk of breast cancer. There were no overall elevations in risk according to the duration of oral contraceptive use, or time intervals since first or last use. Oral contraceptive effects also did not interact with a family history of breast cancer, age at first live birth, use of menopausal oestrogens or obesity. In addition, oral contraceptive use before age 25, before a first birth or during the perimenopausal period did not appear to influence substantially the risk of breast cancer, although there were too few exposed women in some subgroups to assess risk adequately. However, risk estimates for breast cancer in relation to oral contraceptive use did vary according to previous biopsies for benign breast disease and stage of breast cancer at the time of diagnosis.

Previous reports of an adverse effect of oral contraceptives in young women with extended durations of use before age 25 or a first live birth (Paffenbarger, 1977; Pike *et al.*, 1981, 1983; Harris *et al.*, 1982; McPherson *et al.*, 1983, 1987; Olsson *et al.*, 1985; Meirik *et al.*, 1986) prompted us to examine this issue. Since no women in the present study had used oral contraceptives for extended periods of time at an early age, we were unable to estimate risks in relation to such use. However, based on small numbers, oral contraceptive use lasting less than 5 years before age 25 or first birth was not associated with risk.

In contrast to earlier reports (Vessey *et al.*, 1979; Jick *et al.*, 1980; Paffenbarger *et al.*, 1980; Brinton *et al.*, 1982; Henneken *et al.*, 1984), we found no evidence that oral contraceptive use around the time of menopause increased the risk of breast cancer. Premenopausal women who took oral contraceptives for extended periods of time after age 40 were not found to be at high risk. Use of oral contraceptives during the perimenopause might be expected to enhance the risk of breast cancer by prolonging menstrual cycling, maintaining higher levels of oestrogens and progestogens at a time when these hormones would be circulating at lower levels due to changes in ovarian function associated with the menopause.

It is noteworthy that we found no evidence of significant effect modification of oral contraceptives by other breast cancer risk factors. These results fail to confirm earlier studies that found certain high-risk groups of users, specifically those with a family history of breast cancer (Black *et al.*, 1980; Brinton *et al.*, 1982). An earlier report based on a subset of this study population showed elevated risks for users of oral contraceptives who had a positive history of breast cancer in a sister(s) (Brinton *et al.*, 1982). This discrepancy with the present results may be due to the smaller numbers of exposed women included in the initial report, and to the decision to limit this analysis of sister(s) with breast cancer to women who reported having at least one sister.

We did observe elevations in risk among oral contraceptive users with two or more previous biopsies for benign breast lesions. Within the context of the BCDDP, women with two or more breast biopsies are thought to represent the group that is more similar to benign breast disease defined in other studies. In an earlier publication based on participants of the BCDDP, Brinton *et al.* (1979) noted the high prevalence of breast biopsy in this screening population and that a previous history of one biopsy was not a risk factor for breast cancer (RR = 0.83), whereas a history of more than one biopsy was associated with an increased risk (RR = 2.1). These present findings are somewhat surprising since oral contraceptives protect against benign breast disease. It is possible that the benign lesions arising in the context of exposure to oral contraceptives which protect against such lesions represent unusual types that are more strongly related to breast cancer risk.

The relationship between oral contraceptive use and the stage of breast cancer at the time of diagnosis was of primary interest in the present study. Previous studies reported less advanced disease in users, and ascribed the finding to either surveillance bias from early detection of tumours in women taking oral contraceptives or favourable biological effects of oral contraceptives on tumour growth (Vessey et al., 1979, 1983; Ravnihar et al., 1988). Skegg (1988) recently reviewed the potential influence of surveillance bias on results of studies of breast cancer in relation to oral contraceptives, and noted that such bias could produce a spuriously elevated risk. This would be particularly true if oral contraceptive users more frequently practice breast self-examination or have more routine screening by medical personnel (palpation and mammography), resulting in the identification of the breast cancer at an earlier stage.

In addition to the potential influence of surveillance bias, there is some suggestion that oral contraceptives may exert favourable biological activities on tumour growth and spread. Studies of prognosis reported an apparent survival advantage in breast cancer patients with a history of oral contraceptive use (Spencer et al., 1978; Vessey et al., 1979; Matthews et al., 1981; Rosner & Lane, 1986), although adjustment for the stage of disease at diagnosis reduced the beneficial effect in two studies (Vessey et al., 1979; Rosner & Lane, 1986). A third study of oral contraceptives and survival in breast cancer patients failed to support the notion that oral contraceptives confer a positive influence on breast tumour growth (Millard et al., 1987). Further, data from a recent cohort study showed similar 5-year survival rates in oral contraceptive users and controls (Kay & Hannaford, 1988).

Our findings contrast with prior reports (Vessey et al., 1979, 1981; Ravnihar et al., 1988) that noted a higher proportion of oral contraceptive users in women with early stage disease. Although no significant relationships were observed between ever use of oral contraceptives and the clinical stage of disease at diagnosis, dissimilar risk patterns were noted for users of 5 or more years duration among women with *in situ* compared to invasive disease. In the present study, oral contraceptive use was associated with a reduction in risk for *in situ* disease in both recent users and those who discontinued use > 1 year before diagnosis, but a 40–50% increase in risk for invasive cancer. Since all women included in the present study were identified through a breast cancer screening programme, it is less likely that our results can be explained by surveillance bias. In fact, the lower risk estimates observed for *in situ* breast cancer in relation to oral contraceptive use argue against an 'early detection' bias (Skegg, 1988). The results of this study must await confirmation, but suggest that oral contraceptive effects may vary by stage of disease. However, since results based on subgroup analyses may be due to chance alone, these findings should be interpreted cautiously.

Few prior studies have analysed breast cancer risk factors according to stage of disease. Brinton et al. (1983) found generally similar risk factor profiles for women with benign breast disease and those with *in situ* breast cancer; however, different predictors of risk were observed for *in situ* compared to invasive disease. Our findings regarding oral contraceptive effects by pathological stage of disease support the notion that *in situ* and invasive tumours may as groups be aetiologically dissimilar.

In summary, our results provide further evidence against a causal relationship between ever use of oral contraceptives and breast cancer. Women with several prior biopsied benign breast lesions who use oral contraceptives, however, may experience some elevation in risk. The finding that oral contraceptive use of 5 or more years duration is associated with reduced risk for *in situ* disease, but increased risk for invasive cancer must await confirmation. Based on these results, additional studies of oral contraceptives in relation to the stage of breast cancer at the time of diagnosis are needed. Such future studies should account for possible sources of surveillance bias that may influence investigations of oral contraceptives and breast cancer.

### References

BLACK, M.M., KWON, S., LEIS, H.P. & BARCLAY, T.H.C. (1980). Family history and oral contraceptives. *Cancer*, **46**, 2747.

BRESLOW, N.E. & DAY, N.E. (1980). *The Analysis of Case–control Studies*. IARC: Lyon.

BRINTON, L.A., WILLIAMS, R.R., HOOVER, R.N., STEGENS, N.L., FEINLEIB, M. & FRAUMENI, J.F. JR (1979). Breast cancer risk factors among screening program participants. *J. Natl Cancer Inst.*, **62**, 37.

BRINTON, L.A., HOOVER, R.N., SZKLO, M. & FRAUMENI, J.F. JR (1982). Oral contraceptives and breast cancer. *Int. J. Epidemiol.*, **11**, 316.

BRINTON, L.A., HOOVER, R. & FRAUMENI, J.F. JR (1983). Epidemiology of minimal breast cancer. *J. Am. Med. Assoc.*, **249**, 483.

BRINTON, L.A., SCHAIRER, C., STANFORD, J.L. & HOOVER, R.N. (1986*a*). Cigarette smoking and breast cancer. *Am. J. Epidemiol.*, **123**, 614.

BRINTON, L.A., HOOVER, R. & FRAUMENI, J.F. JR (1986*b*). Menopausal oestrogens and breast cancer risk: an expanded case–control study. *Br. J. Cancer*, **54**, 825.

CENTERS FOR DISEASE CONTROL (1983). Long-term oral contraceptive use and the risk of breast cancer. *J. Am. Med. Assoc.*, **249**, 1591.

FASAL, E. & PAFFENBARGER, R.S. (1975). Oral contraceptives as related to cancer and benign lesions of the breast. *J. Natl Cancer Inst.*, **55**, 767.

HARRIS, N.V., WEISS, N.S., FRANCIS, A.M. & POLISSAR, L. (1982). Breast cancer in relation to patterns of oral contraceptive use. *Am. J. Epidemiol.*, **116**, 643.

HENDERSON, B.E., ROSS, R.K., PIKE, M.C. & CASAGRANDE, J.T. (1982). Endogenous hormones as a major factor in human cancer. *Cancer Res.*, **42**, 3232.

HENNEKEN, C.H., SPEIZER, F.E., LIPNICK, R.J. and 6 others (1984). A case–control study of oral contraceptive use and breast cancer. *J. Natl Cancer Inst.*, **72**, 39.

JANERICH, D.T., POLEDNAK, A.P., GLEBATIS, D.M. & LAWRENCE, C.E. (1983). Breast cancer and oral contraceptive use: a case–control study. *J. Chron. Dis.*, **36**, 639.

JICK, H., WALKER, A.M., WATKINS, R.N. and 6 others (1980). Oral contraceptives and breast cancer. *Am. J. Epidemiol.*, **112**, 577.

KAY, C. (1981). Breast cancer and oral contraceptives: findings in Royal College of General Practitioners' Study. *Br. Med. J.*, **282**, 2089.

KAY, C.R. & HANNAFORD, P.C. (1988). Breast cancer and the pill – a further report from the Royal College of General Practitioners' oral contraception study. *Br. J. Cancer*, **58**, 675.

KELSEY, J.L., FISCHER, D.B., HOLFORD, T.R. and 4 others (1981). Exogenous estrogens and other factors in the epidemiology of breast cancer. *J. Natl Cancer Inst.*, **67**, 327.

KELSEY, J.L. & HILDRETH, N.G. (1983). *Breast and Gynecologic Cancer Epidemiology*. CRC Press: Boca Raton, Florida.

LA VECCHIA, C., DECARLI, A., FASOLI, M. and 5 others (1986). Oral contraceptives and cancers of the breast and of the female genital tract. Interim results from a case–control study. *Br. J. Cancer*, **54**, 311.

LEES, A.W., BURNS, P.E. & GRACE, M. (1978). Oral contraceptives and breast disease in premenopausal northern Albertan women. *Int. J. Cancer*, **22**, 700.

LIPNICK, R.J., BURING, J.E., HENNEKEN, C.H. and 7 others. (1986). Oral contraceptives and breast cancer. *J. Am. Med. Assoc.*, **255**, 58.

LUBIN, J. (1981). A computer program for the analysis of matched case–control studies. *Comput. Biomed. Res.*, **14**, 138.

MATTHEWS, P.N., MILLIS, R.R. & HAYWARD, J.L. (1981). Breast cancer in women who have taken contraceptive steroids. *Br. Med. J.*, **282**, 774.

McPHERSON, K., NEIL, A., VESSEY, M.P. & DOLL, R. (1983). Oral contraceptives and breast cancer. *Lancet*, **ii**, 1414.

McPHERSON, K., VESSEY, M.P., NEIL, A., DOLL, R., JONES, L. & ROBERTS, M. (1987). Early oral contraceptive use and breast cancer: results of another case–control study. *Br. J. Cancer*, **56**, 653.

MEIRIK, O., ADAMI, H.O., CHRISTOFFERSEN, T., LUND, E., BERGSTROM, R. & BERGSJO, P. (1986). Oral contraceptive use and breast cancer in young women. *Lancet*, **ii**, 650.

MILLARD, F.C., BLISS, J.M., CHILVERS, C.E.D. & GAZET, J.C. (1987). Oral contraceptives and survival in breast cancer. *Br. J. Cancer*, **56**, 377.

MILLER, D.R., ROSENBERG, L., KAUFMAN, D.W., SCHOTTENFELD, D., STOLLEY, P.D. & SHAPIRO, S. (1986). Breast cancer risk in relation to early oral contraceptive use. *Obstet. Gynecol.*, **68**, 863.

OLSSON, H., OLSSON, M.L., MOLLER, T.R., RANSTAM, R. & HOLM, P. (1985). Oral contraceptive use and breast cancer in young women in Sweden. *Lancet*, **i**, 748.

PAFFENBARGER, R.S., FASAL, E., SIMMONS, M.E. & KAMPERT, J.B. (1977). Cancer risk as related to use of oral contraceptives during fertile years. *Cancer*, **39**, 1887.

PAFFENBARGER, R.S., KAMPERT, J.B. & CHANG, H.-G. (1980). Characteristics that predict risk of breast cancer before and after the menopause. *Am. J. Epidemiol.*, **112**, 258.

PAUL, C., SKEGG, D.C.G., SPEARS, G.F.S. & KALDOR, J.M. (1986). Oral contraceptives and breast cancer: a national study. *Br. Med. J.*, **293**, 723.

PIKE, M.C., HENDERSON, B.E., CASAGRANDE, J.T., ROSARIO, I. & GRAY, G.E. (1981). Oral contraceptive use and early abortion as risk factors for breast cancer in young women. *Br. J. Cancer*, **43**, 72.

PIKE, M.C., KRAILO, M.D., HENDERSON, B.E., DUKE, A. & ROY, S. (1983). Breast cancer in young women and use of oral contraceptives: possible modifying effect of formulation and age at use. *Lancet*, **ii**, 926.

PRENTICE, R.L. & THOMAS, D.B. (1987). On the epidemiology of oral contraceptives and disease. *Adv. Cancer Res.*, **49**, 285.

RAVNIHAR, B., ZAKELJ, P., KOSMELJ, K. & STARE, J. (1988). A case–control study of breast cancer in relation to oral contraceptive use in Slovenia. *Neoplasma*, **35**, 109.

ROSENBERG, L., MILLER, D.R., KAUFMAN, D.W. and 4 others. (1984). Breast cancer and oral contraceptive use. *Am. J. Epidemiol.*, **119**, 167.

ROSNER, D. & LANE, W.W. (1986). Oral contraceptive use has no adverse effect on the prognosis of breast cancer. *Cancer*, **57**, 591.

SATTIN, R.W., RUBIN, G.L., WINGO, P.A., WEBSTER, L.A. & ORY, H.W. (1986). Oral-contraceptive use and the risk of breast cancer. *N. Engl. J. Med.*, **315**, 405.

SCHLESSELMAN, J.J., STADEL, B.S., MURRAY, P. & LAI, S. (1988). Breast cancer in relation to early use of oral contraceptives – no evidence of a latent effect. *J. Am. Med. Assoc.*, **259**, 1828.

SKEGG, D.C.G. (1988). Potential for bias in case–control studies of oral contraceptives and breast cancer. *Am. J. Epidemiol.*, **127**, 205.

SPENCER, J.D., MILLIS, R.R. & HAYWARD, J.L. (1978). Contraceptive steroids and breast cancer. *Br. Med. J.*, **i**, 1024.

STADEL, B.V., WEBSTER, L.A., RUBIN, G.L., SCHLESSELMAN, J.J. & WINGO, P.A. (1985). Oral contraceptives and breast cancer in young women. *Lancet*, **ii**, 970.

TRAPIDO, E.J. (1981). A prosepctive cohort study or oral contraceptives and breast cancer. *J. Natl Cancer Inst.*, **67**, 1011.

VESSEY, M.P., DOLL, R., JONES, K., McPHERSON, K. & YEATES, D. (1979). An epidemiological study of oral contraceptives and breast cancer. *Br. Med. J.*, **i**, 1755.

VESSEY, M.P., McPHERSON, K. & DOLL, R. (1981). Breast cancer and oral contraceptives: findings in Oxford – Family Planning Association contraceptive study. *Br. Med J.*, **282**, 2093.

VESSEY, M.P., McPHERSON, K., YEATES, D. & DOLL, R. (1982). Oral contraceptive use and abortion before first term pregnancy in relation to breast cancer risk. *Br. J. Cancer*, **45**, 327.

VESSEY, M., BARON, J., DOLL, R., McPHERSON, K. & YEATES, D. (1983). Oral contraceptives and breast cancer: final report of an epidemiological study. *Br. J. Cancer*, **47**, 455.